# Supplementary figures and images for: Differential roles of the type I and II secretion systems for the intracellular ABC141 Acinetobacter baumannii infection, which elicits an atypical hypoxia response in endothelial cells
Source: PLoS Pathog. 2026 Feb 9;22(2):e1013265. doi: 10.1371/journal.ppat.1013265 (PMC12912691; doi:10.1371/journal.ppat.1013265)

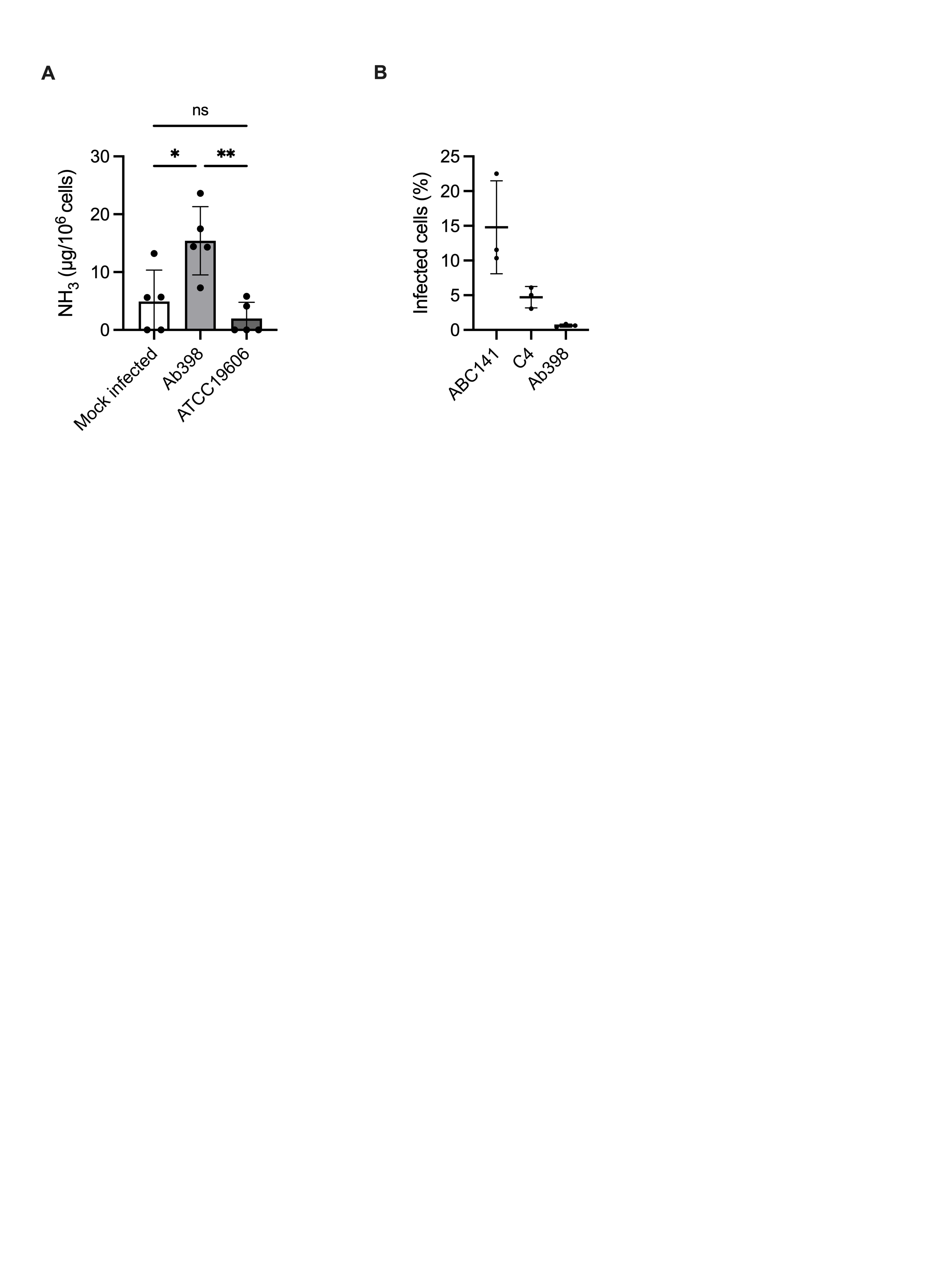

Supplement: S1 Fig — (A) Quantification of the level of ammonia in cells either mock, Ab398 or ATCC19606 infected RAW macrophage-like cells for 6 h, normalized to the total number of cells for each condition. Data correspond to the means ± SD of 5 independent experiments. Comparisons were made with a One Way ANOVA with Tukey’s correction with * indicating P < 0.05; ** P < 0.01, and ns non-significant. (B) Quantification of the percentage of infected cells at 2 h post-infection of endothelial EA.hy cells with either ABC141, C4 or Ab398. Data correspond to the means ± SD of 3 independent experiments. (TIFF) [file ppat.1013265.s001.tiff]

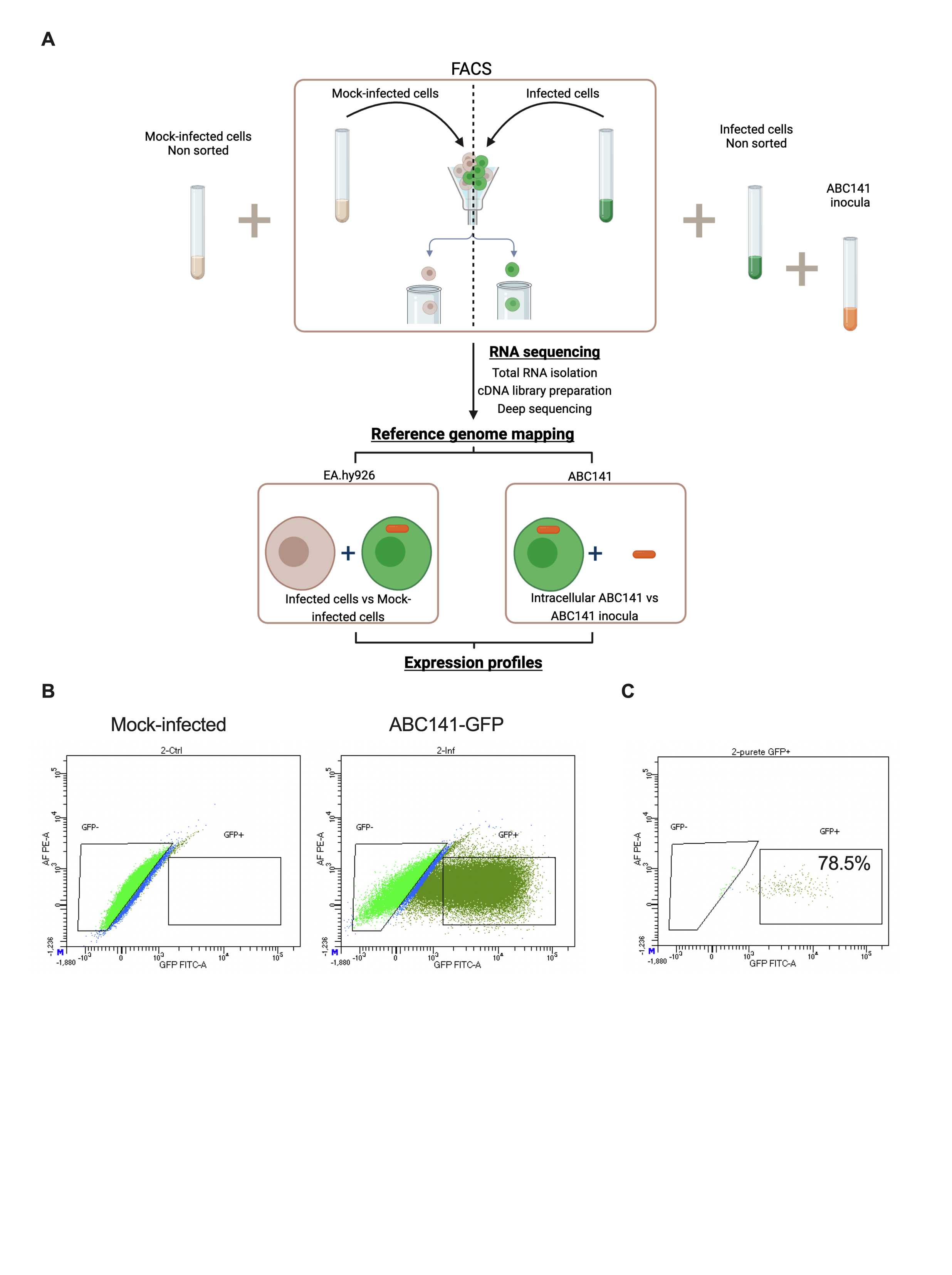

Supplement: S2 Fig — (A) Diagram describing the experimental strategy for the Dual-RNAseq experiment. Endothelial cells were infected for 24h with GFP-expressing ABC141 A. baumannii. RNA was extracted from the inocula, from infected cells and from control mock-infected cells. In addition, a separate experiment was carried out by sorting GFP-infected cells. A control mock-infected sample was also included. This experimental set up was used in 4 independent experiments; RNA extracted and sequencing done in all samples. Diagram created in BioRender. Salcedo, S. (2025) https://urldefense.com/v3/__https://BioRender.com/fje8ay8__;!!Mak6IKo!JlWeSU0KqKNjs0yM2N0-KcaBim4P-xD2P1MVZ1SE2oKihzJcAhBUiCRle1S5I9nxnzilZdXyYGDZCXn1$. (B) Gating strategy for sorting of mock infected cells (left, (GFP-) and GFP-infected cells (right, GFP+). (C) Level of purity of a represented sorted sample with 78.5% sorted cells positive for GFP. (TIFF) [file ppat.1013265.s002.tiff]

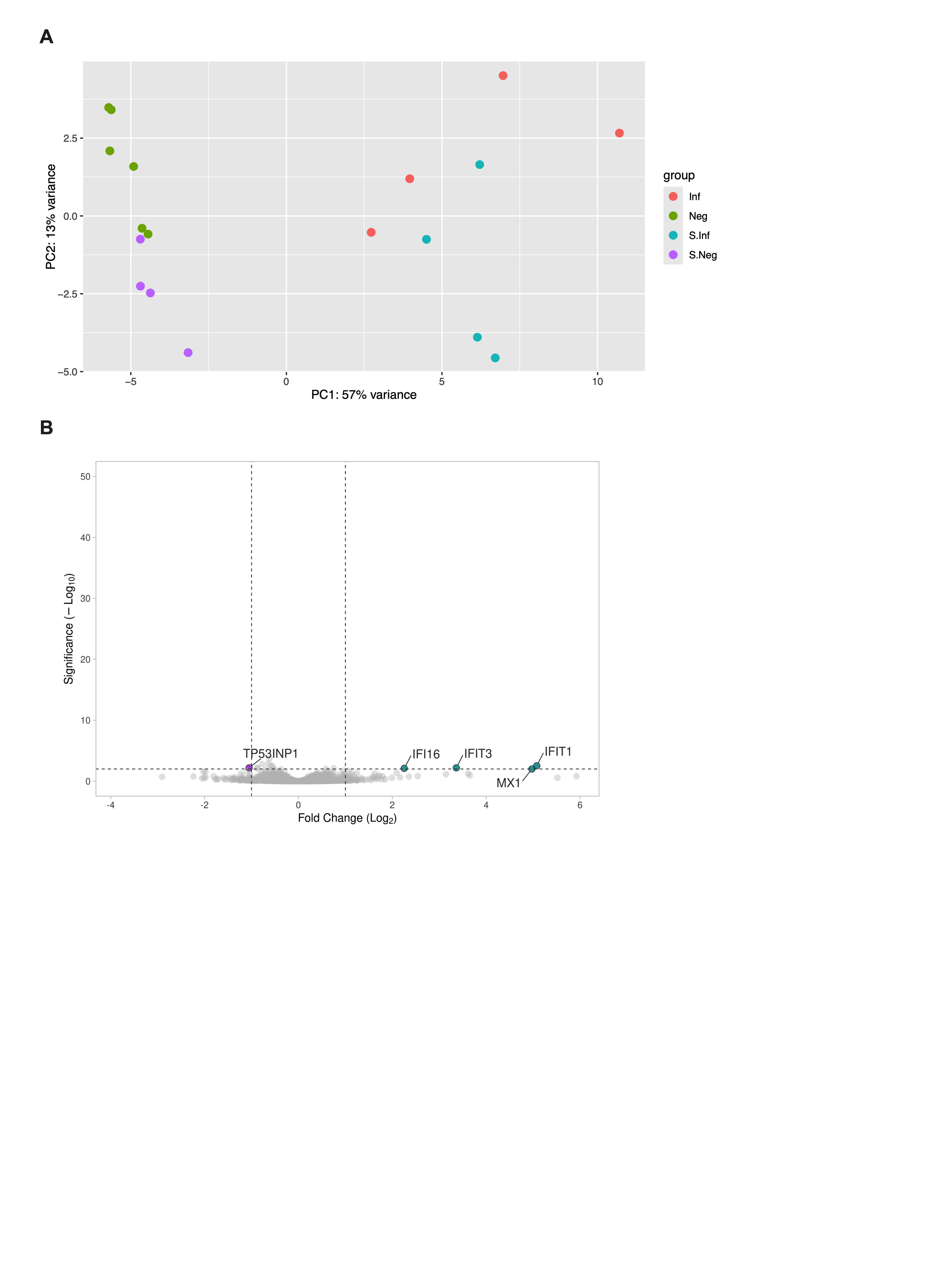

Supplement: S3 Fig — (A) Principal Component Analysis (PCA) plot showing the variance in eukaryotic gene expression profiles between mock-infected cells (Neg; green), sorted mock-infected cells (SNeg; purple), ABC141-GFP infected cells (Inf; red) and sorted ABC141-GFP infected cells (SInf; blue). Infections were done for 24h. Each point represents a biological replicate, and the axes indicate the percentage of variance explained by the first two principal components. (B) Volcano plot showing differential host gene expression of sorted mock-infected versus unsorted mock-infected cells. The x-axis represents the log₂ fold change, and the y-axis represents the –log₁₀ adjusted p-value. Human genes with log₂ fold change ≥ 1 and adjusted p-value < 0.01 are considered upregulated (cyan). (TIFF) [file ppat.1013265.s003.tiff]

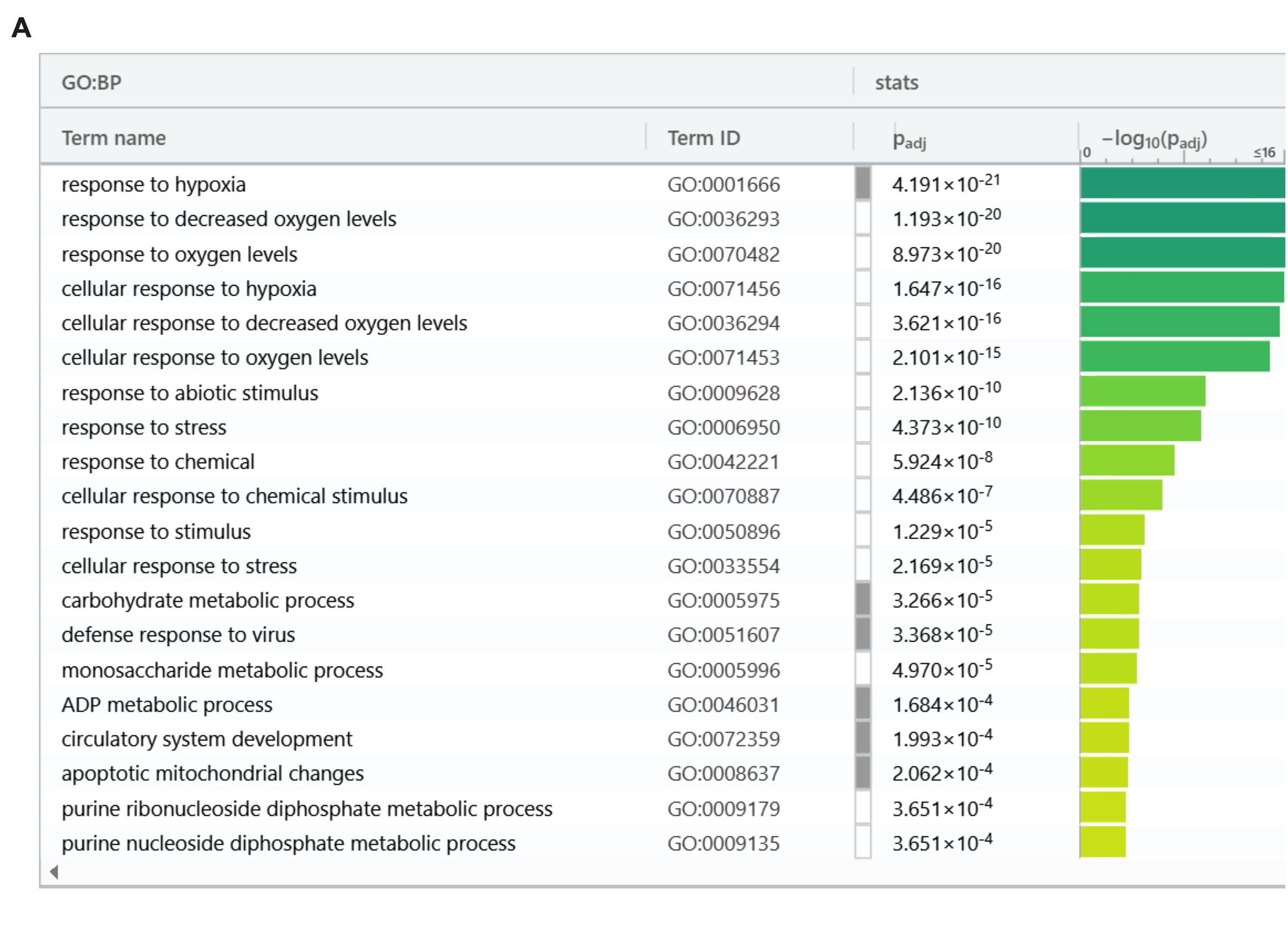

Supplement: S4 Fig — (A) Gene Ontology (GO) enrichment analysis of host genes associated with upregulated genes. Enriched GO terms in the Biological Process category are shown with p-value < 0.01. Only top 20 GO terms are shown. (TIFF) [file ppat.1013265.s004.tiff]

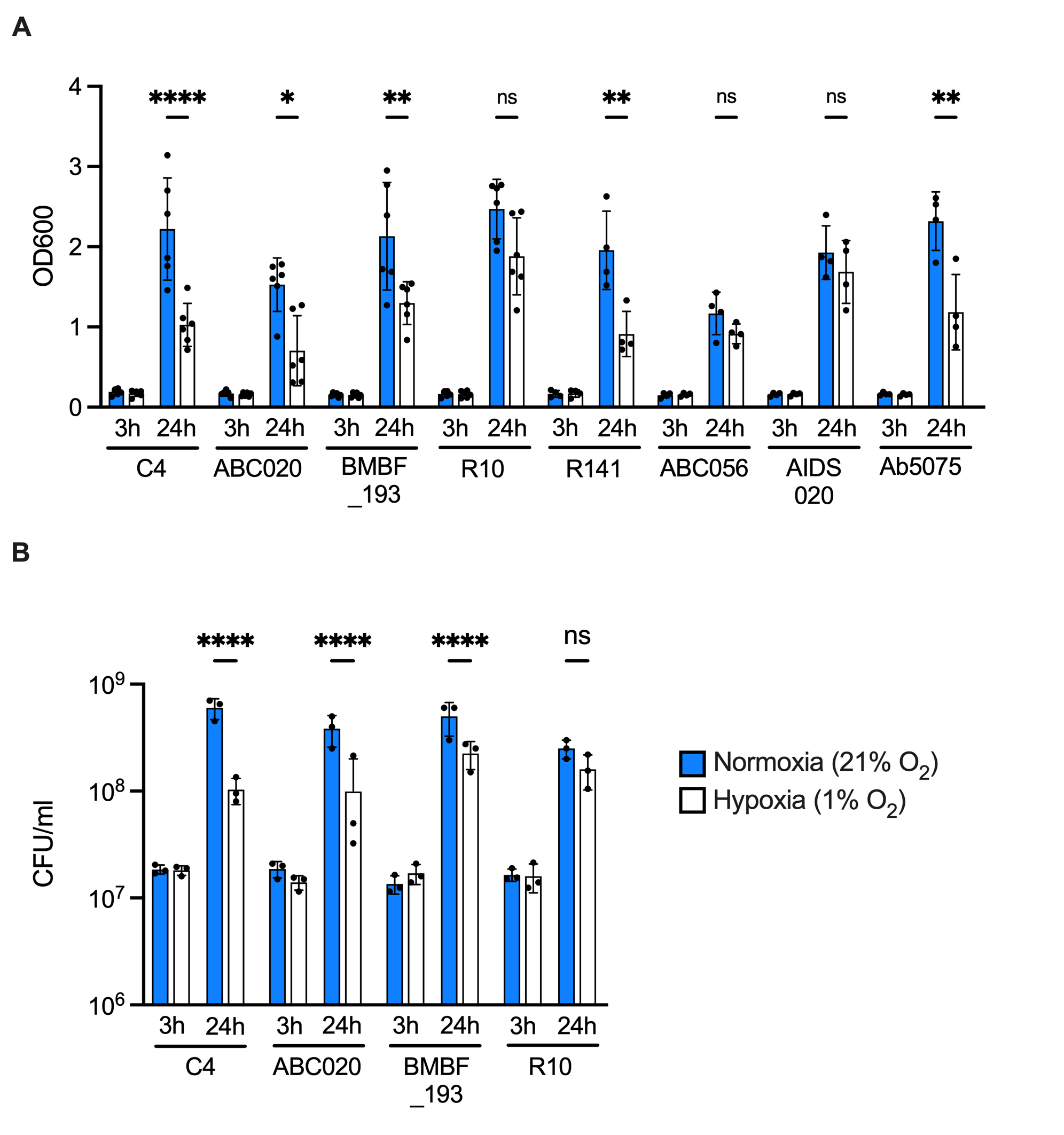

Supplement: S5 Fig — (A) Quantification of the OD600 or (B) CFU of different A. baumannii cultures incubated in either normoxia (20% O2, blue bars) or hypoxia (1% O2, white bars) for 3 and 24h. Data are means ± SD, N = 4. Comparisons were done with a One Way ANOVA with * indicating P < 0.05, ** P < 0.01, **** P < 0.0001 and “ns” non-significant. Strains C4, ABC020, BMBF_193 and R10 have been shown to be able to multiply inside non-phagocytic cells unlike strains R141, ABC056, AIDS020 and Ab5075. (TIFF) [file ppat.1013265.s005.tiff]

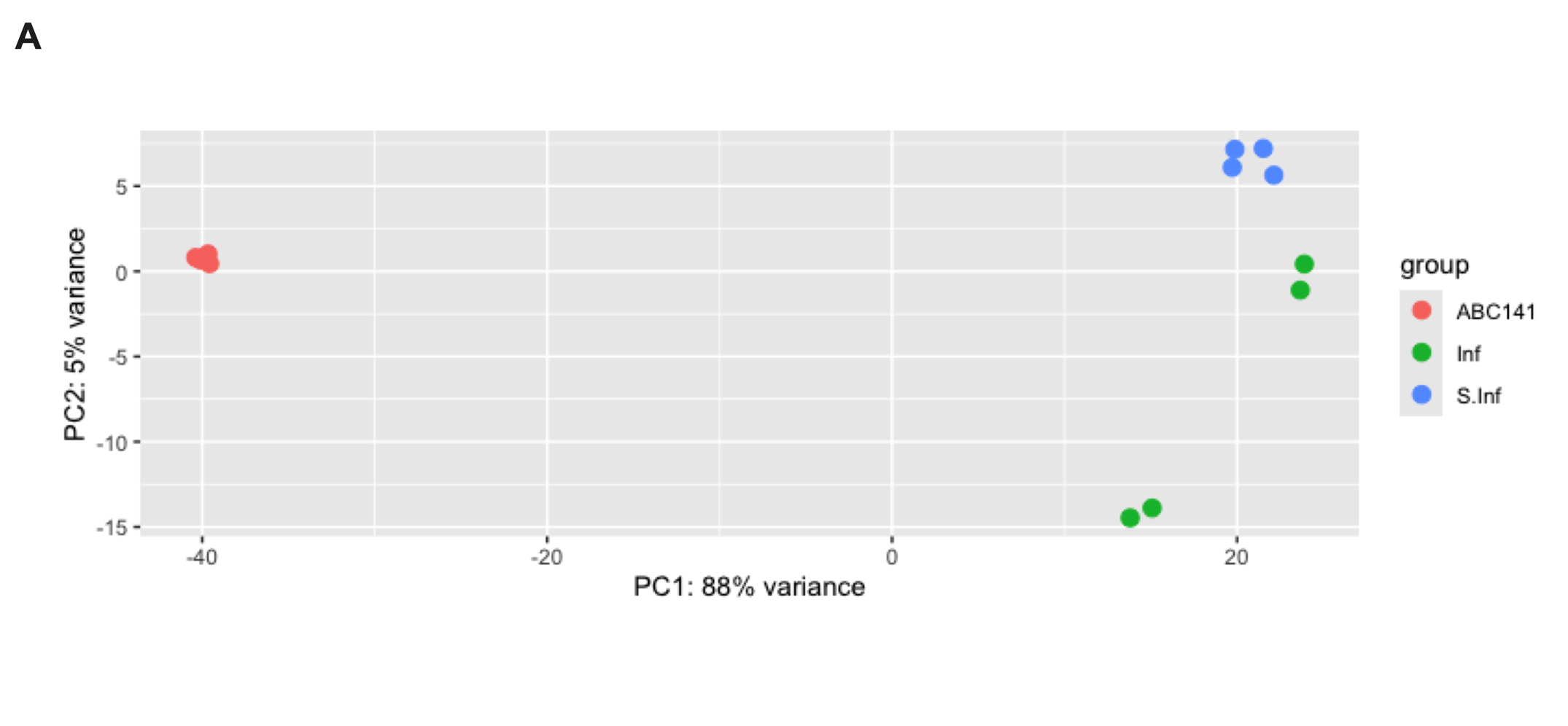

Supplement: S6 Fig — Each point represents a biological replicate, and the axes indicate the percentage of variance explained by the first two principal components. (TIFF) [file ppat.1013265.s006.tiff]

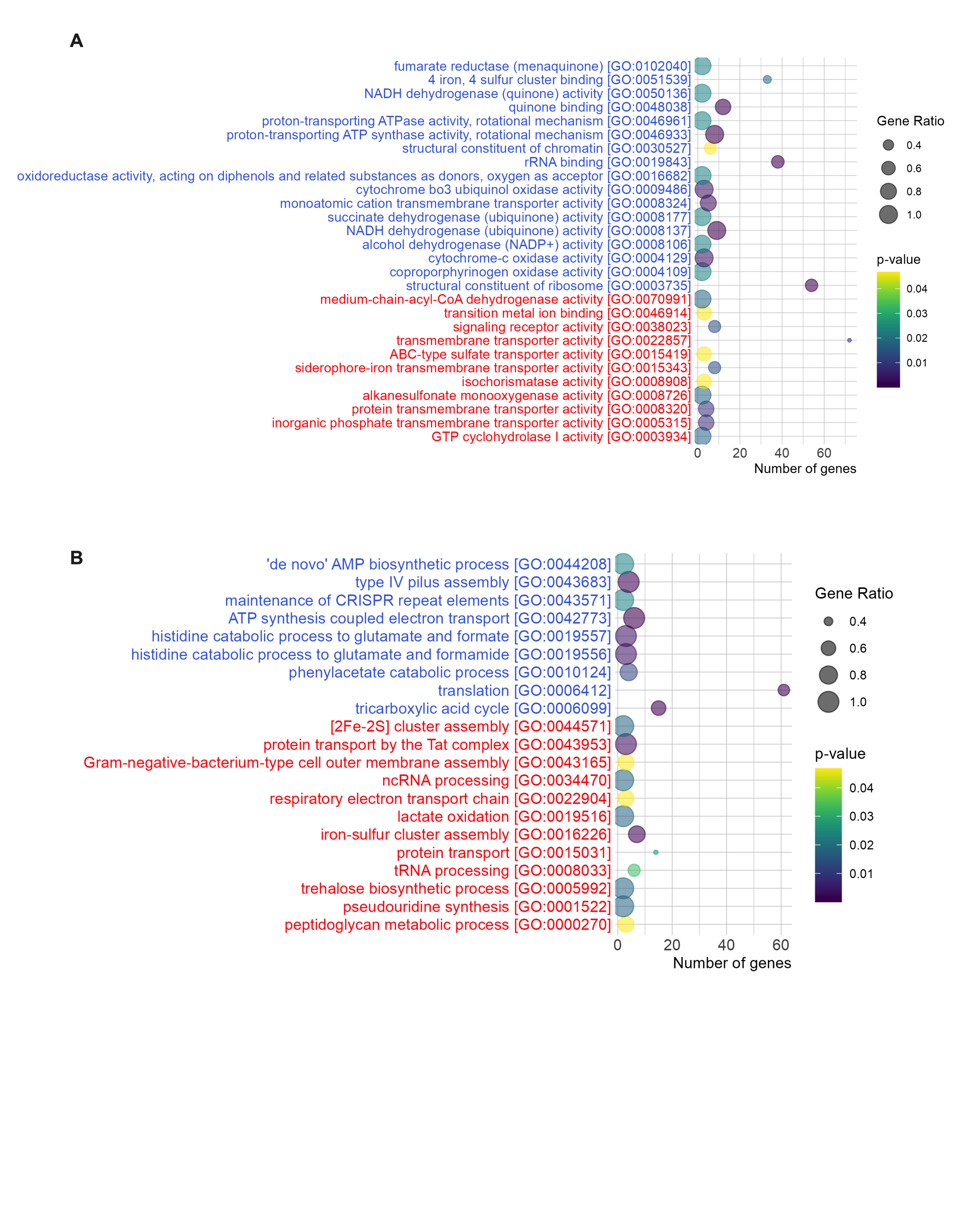

Supplement: S7 Fig — Enriched GO terms in the (A) Molecular function or (B) Biological Process category are shown with p-value < 0.05. Terms associated with upregulated genes are labeled in red font, and those associated with downregulated genes are labeled in blue font. (TIFF) [file ppat.1013265.s007.tiff]

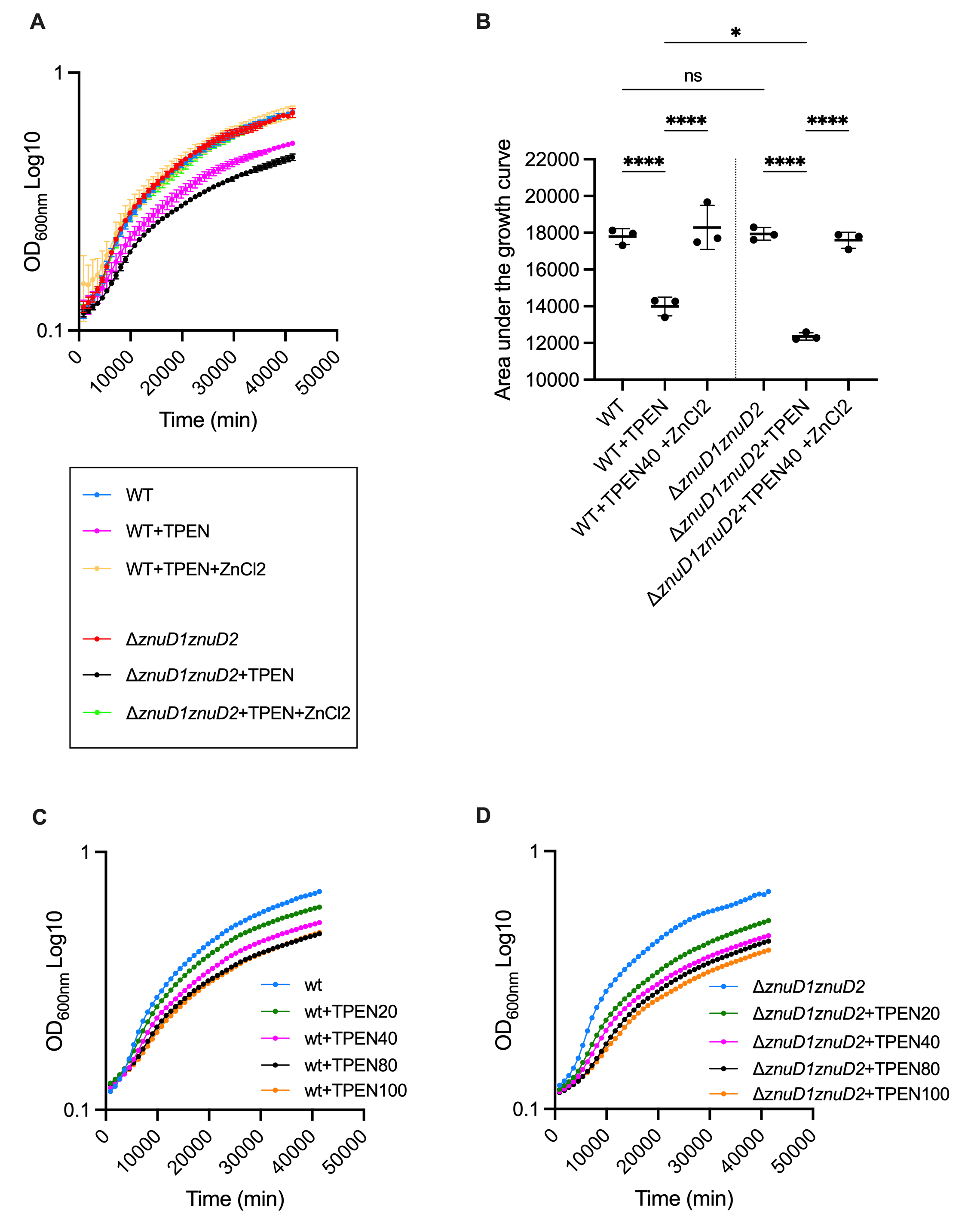

Supplement: S8 Fig — The wild-type (WT) and the ∆znuD1 ∆znuD2 mutant strains were grown in LB or LB with 40 µM of TPEN zinc chelator, with or without 40 µM of ZnCl2. (A) The OD600 was monitored over time in 3 independent experiments (data correspond to means ± SD). (B) The area under the curve was calculated including for WT and mutant grown in the presence of 40 µM of TPEN and supplemented with 40 µM of ZnCl2. (C) The wild-type ABC141 and (D) the ∆znuD1 ∆znuD2 mutant were grown in increasing concentrations of TPEN, from 20 µM to 100 µM. (TIFF) [file ppat.1013265.s008.tiff]
