# Supplementary material for: Differential roles of the type I and II secretion systems for the intracellular ABC141 Acinetobacter baumannii infection, which elicits an atypical hypoxia response in endothelial cells
Source: PLoS Pathog. 2026 Feb 9;22(2):e1013265. doi: 10.1371/journal.ppat.1013265 (PMC12912691; doi:10.1371/journal.ppat.1013265)
Supplement: S2 Table — Enriched GO terms in the Biological Process category are shown with p-value < 0.01. Only top 20 Go terms are shown. (DOCX) [file ppat.1013265.s010.docx]

| **GO:BP** | | **Stats** | |
| --- | --- | --- | --- |
| **Term name** | **Term ID** | **P_adj_** | **- log_10_(P_adj_)** |
| Response to hypoxia | GO:0001666 | 4.191.10^-21^ | 20.38 |
| Response to decreased oxygen levels | GO:0036293 | 1.193.10^-20^ | 19.92 |
| Response to oxygen levels | GO:0070482 | 8.973.10^-20^ | 19.05 |
| Cellular response to hypoxia | GO:0071456 | 1.647.10^-16^ | 15.78 |
| Cellular response to decreased oxygen levels | GO:0036294 | 3.621.10^-16^ | 15.44 |
| Cellular response to oxygen levels | GO:0071453 | 2.101.10^-15^ | 14.68 |
| Response to abiotic stimulus | GO:0009628 | 2.136.10^-10^ | 9.67 |
| Response to stress | GO:0006950 | 4.373.10^-10^ | 9.36 |
| Response to chemical | GO:0042221 | 5.924.10^-8^ | 7.23 |
| Cellular response to chemical stimulus | GO:0070887 | 4.486.10^-7^ | 6.35 |
| Response to stimulus | GO:0050896 | 1.229.10^-5^ | 4.91 |
| Cellular response to stress | GO:0033554 | 2.169.10^-5^ | 4.66 |
| Carbohydrate metabolic process | GO:0005975 | 3.266.10^-5^ | 4.49 |
| Defense response to virus | GO:0051607 | 3.368.10^-5^ | 4.47 |
| Monosaccharide metabolic process | GO:0005996 | 4.970.10^-5^ | 4.31 |
| ADP metabolic process | GO:0046031 | 1.684.10^-4^ | 3.77 |
| Circulatory system development | GO:0072359 | 1.993.10^-4^ | 3.70 |
| Apoptic mitochondrial changes | GO:0008637 | 2.062.10^-4^ | 3.69 |
| Purine ribonucleoside diphosphate metabolic process | GO:0009179 | 3.651.10^-4^ | 3.44 |
| Purine nucleoside diphosphate metabolic process | GO:0009135 | 3.651.10^-4^ | 3.44 |
