# Supplementary material for: Differential roles of the type I and II secretion systems for the intracellular ABC141 Acinetobacter baumannii infection, which elicits an atypical hypoxia response in endothelial cells
Source: PLoS Pathog. 2026 Feb 9;22(2):e1013265. doi: 10.1371/journal.ppat.1013265 (PMC12912691; doi:10.1371/journal.ppat.1013265)
Supplement: S3 Table — (DOCX) [file ppat.1013265.s011.docx]

**S3 Table.** List of hypoxia-related genes identified in the DualRNASeq.

| **Gene** | **Log2 Fold Change** | **Function** | **Roles in hypoxia** | **Reference** |
| --- | --- | --- | --- | --- |
| Carbonic Anhydrase 9 (CA9) | 6.28 | Catalyzes carbon dioxide hydration  pH regulation  Cell-cell adhesion | Induced by HIF-1  Involved in pH regulation during hypoxia | ^1–3^ |
| Hexokinase 2  (HK2) | 5.70 | Phosphorylation of glucose to glucose-6-phosphate  Glycolysis  Pentose phosphate pathway | Induced by HIF-1  Glycolysis and lactate secretion  Anti apoptotic / autophagic role | ^4^ |
| Angiopoietin like 4  (ANGPTL4) | 4.31 | Serum hormone involved in lipid regulation, glucose metabolism and insulin sensitivity | Induced by HIF-1  Pro-angiogenic response  Cell growth promotion | ^5,6^ |
| N-myc Downstream Regulated Gene 1  (NDRG1) | 3.29 | Cell growth  Differentiation  Hormone and stress response  P53 mediated apoptosis | Induced by HIF-1  Cellular adaptation to hypoxia | ^7–10^ |
| egl-9 family hypoxia inducible factor 3  (EGLN3) | 3.02 | Enables peptidyl-proline 4-dioxygenase activity | Induced by HIF-1  Limits physiological activation of HIF-1  Limits glycolysis | ^11,12^ |
| Stanniocalcin 1  (STC1) | 2.92 | SUMO E3 ubiquitin ligase activity | Induced by HIF-1  Glycolysis Regulation  Oxidative stress | ^13,14^ |
| Pyruvate Dehydrogenase Kinase 1  (PDK1) | 2.54 | Catalyzes oxidative decarboxylation of pyruvate | Induced by HIF1  Glycolysis maintenance | ^15–17^ |
| Stanniocalcin 2  (STC2) | 2.33 | Phosphate and calcium regulation  Angiogenesis stimulation | Induced by HIF-1  Cell proliferation | ^18,19^ |
| Adrenomedullin  (ADM) | 1.90 | Inflammatory response  Apoptosis inhibition and ROS reduction | Induced in hypoxia  Angiogenesis  Cell survival | ^20–22^ |
| BCL2 interacting protein 3 like  (BNIP3L/Nix) | 1.83 | Pro apoptotic factor  Mitophagy receptor | Induced by HIF-1  Autophagy / mitophagy induction | ^23–26^ |
| Ankyrin Repeat Domain 37  (ANKRD37) | 1.70 | Regulates NF-κB | Induced by HIF-1  Autophagy induction  Cell growth | ^27,28^ |
| Hypoxia inducible lipid droplet associated  (HILPDA) | 1.70 | Fat storage  Signaling receptor binding activity | Induced by HIF  Regulation of lipid droplets formation | ^29–31^ |
| Family With Sequence Similarity 162 Member A  (FAM162A) | 1.48 |  | Induced by HIF  Promotion of intrinsic apoptosis | ^32^ |
| Adenylate kinase 4  (AK4) | 1.46 | Energy metabolism and mitochondrial homeostasis  Cellular nucleotides homeostasis | Induced by HIF  Hypoxia tolerance  Enhancement of HIF-1α stability  ROS increase | ^33–38^ |
| DNA damage inducible transcript 4  (DDIT4) | 1.43 | Negative regulation of mTOR  responses to cellular energy levels and cellular stress | Induced by HIF-1  HIF-1 negative regulator  ROS production  Negative regulation of mTOR | ^39–44^ |
| BCL2 interacting protein 3  (BNIP3) | 1.33 | Pro-Apoptotic factor | Induced by HIF  Caspase independent apoptosis  Mitophagy | ^26,45–47^ |
| Cbp/P300-Interacting Transactivator, With Glu/Asp Rich Carboxy-Terminal Domain 2  (CITED2) | 1.32 | Links TFAP2 transcription factors and the p300/CBP transcriptional coactivator complex | Induced by HIF  Negative regulator of HIF1α | ^48–50^ |
| Procollagen-Lysine,2-Oxoglutarate 5-Dioxygenase 2  (PLOD2) | 1.32 | Hydroxylation of lysyl residues in collagen-like peptides | Induced by HIF  Cell metabolism and extracellular matrix reorganization | ^51–53^ |
| Solute carrier family 2 member 1  (SLC2A1 or GLUT1) | 1.21 | Major glucose transporter and other aldoses | Induced by HIF | ^54,55^ |
| Egl-9 family Hypoxia inducible factor 1 / prolyl hydroxylase domain containing protein 2  (EGLN1 / PHD2) | 1.18 | Post-translational formation of 4-hydorxyproline in HIFa proteins | Induced by HIF  HIF1α transcriptional activity repression | ^56,57^ |
| Vascular endothelial growth factor A  (VEGFA) | 1.16 | Proliferation and migration  Angiogenesis | Cell death protection  Induction of key genes involved in hypoxia  Angiogenesis | ^58–60^ |
| Phosphoglycerate kinase 1  (PGK1) | 1.12 | Conversion of 1,3-diphosphoglycerate to 3-phosphoglycerate | Induce by HIF  Positive feedback loop  Glycolysis and TCA cycle | ^61–64^ |
| Endoplasmic reticulum oxidoreductase 1 alpha  (ERO1A) | 1.06 | Oxidoreductase activity  Cell redox homeostasis | Induced by HIF  Integrin glycosylation  Membrane transport  Angiogenesis | ^65–68^ |
| Nucleolar Protein 3  (NOL3) | 1.06 | Anti apoptotic protein | Induced by HIF  Proliferation and cell death inhibition | ^69–71^ |

1. Wykoff, C. C. *et al.* Hypoxia-inducible expression of tumor-associated carbonic anhydrases. *Cancer Res.* 60, 7075–83 (2000).

2. Sedlakova, O. *et al.* Carbonic anhydrase IX, a hypoxia-induced catalytic component of the pH regulating machinery in tumors. *Front. Physiol.* 4, 400 (2014).

3. Švastová, E. *et al.* Hypoxia activates the capacity of tumor‐associated carbonic anhydrase IX to acidify extracellular pH. *FEBS Lett.* 577, 439–445 (2004).

4. Ikeda, S. *et al.* Hypoxia‐inducible hexokinase‐2 enhances anti‐apoptotic function via activating autophagy in multiple myeloma. *Cancer Sci.* 111, 4088–4101 (2020).

5. Baba, K. *et al.* Hypoxia-induced ANGPTL4 sustains tumour growth and anoikis resistance through different mechanisms in scirrhous gastric cancer cell lines. *Sci. Rep.* 7, 11127 (2017).

6. Tan, M. J., Teo, Z., Sng, M. K., Zhu, P. & Tan, N. S. Emerging Roles of Angiopoietin-like 4 in Human Cancer. *Mol. Cancer Res.* 10, 677–688 (2012).

7. Cangul, H. Hypoxia upregulates the expression of the NDRG1 gene leading to its overexpression in various human cancers. *BMC Genet.* 5, 27 (2004).

8. Cai, K., El-Merahbi, R., Loeffler, M., Mayer, A. E. & Sumara, G. Ndrg1 promotes adipocyte differentiation and sustains their function. *Sci. Rep.* 7, 7191 (2017).

9. Sheng, X., Li, X., Qian, Y., Wang, S. & Xiao, C. ETS1 regulates NDRG1 to promote the proliferation, migration, and invasion of OSCC. *Oral Dis.* 30, 977–990 (2024).

10. Stein, S. *et al.* NDRG1 Is Necessary for p53-dependent Apoptosis*. *J. Biol. Chem.* 279, 48930–48940 (2004).

11. Strocchi, S., Reggiani, F., Gobbi, G., Ciarrocchi, A. & Sancisi, V. The multifaceted role of EGLN family prolyl hydroxylases in cancer: going beyond HIF regulation. *Oncogene* 41, 3665–3679 (2022).

12. Chen, N. *et al.* The oxygen sensor PHD3 limits glycolysis under hypoxia via direct binding to pyruvate kinase. *Cell Res.* 21, 983–986 (2011).

13. Sun, B. *et al.* Stanniocalcin-1 Protected Astrocytes from Hypoxic Damage Through the AMPK Pathway. *Neurochem. Res.* 46, 2948–2957 (2021).

14. Yeung, H. Y. *et al.* Hypoxia-Inducible Factor-1-Mediated Activation of Stanniocalcin-1 in Human Cancer Cells. *Endocrinology* 146, 4951–4960 (2005).

15. Papandreou, I., Cairns, R. A., Fontana, L., Lim, A. L. & Denko, N. C. HIF-1 mediates adaptation to hypoxia by actively downregulating mitochondrial oxygen consumption. *Cell Metab.* 3, 187–197 (2006).

16. Wigfield, S. M. *et al.* PDK-1 regulates lactate production in hypoxia and is associated with poor prognosis in head and neck squamous cancer. *Br. J. Cancer* 98, 1975–1984 (2008).

17. Semba, H. *et al.* HIF-1α-PDK1 axis-induced active glycolysis plays an essential role in macrophage migratory capacity. *Nat. Commun.* 7, 11635 (2016).

18. Law, A. Y. S. & Wong, C. K. C. Stanniocalcin-2 is a HIF-1 target gene that promotes cell proliferation in hypoxia. *Exp. Cell Res.* 316, 466–476 (2010).

19. Zeiger, W. *et al.* Stanniocalcin 2 Is a Negative Modulator of Store-Operated Calcium Entry. *Mol. Cell. Biol.* 31, 3710–3722 (2011).

20. Kim, S.-M., Kim, J.-Y., Lee, S. & Park, J.-H. Adrenomedullin protects against hypoxia/reoxygenation‐induced cell death by suppression of reactive oxygen species via thiol redox systems. *FEBS Lett.* 584, 213–218 (2010).

21. Kato, H., Shichiri, M., Marumo, F. & Hirata, Y. Adrenomedullin as an Autocrine/Paracrine Apoptosis Survival Factor for Rat Endothelial Cells. *Endocrinology* 138, 2615–2620 (1997).

22. Kitamura, K. *et al.* Adrenomedullin: A Novel Hypotensive Peptide Isolated from Human Pheochromocytoma. *Biochem. Biophys. Res. Commun.* 192, 553–560 (1993).

23. Li, Y. *et al.* BNIP3L/NIX-mediated mitophagy: molecular mechanisms and implications for human disease. *Cell Death Dis.* 13, 14 (2021).

24. Novak, I. *et al.* Nix is a selective autophagy receptor for mitochondrial clearance. *EMBO Rep.* 11, 45–51 (2010).

25. Bellot, G. *et al.* Hypoxia-Induced Autophagy Is Mediated through Hypoxia-Inducible Factor Induction of BNIP3 and BNIP3L via Their BH3 Domains. *Mol. Cell. Biol.* 29, 2570–2581 (2009).

26. Zhang, J. & Ney, P. A. Role of BNIP3 and NIX in cell death, autophagy, and mitophagy. *Cell Death Differ.* 16, 939–946 (2009).

27. Deng, M., Zhang, W., Yuan, L., Tan, J. & Chen, Z. HIF-1a regulates hypoxia-induced autophagy via translocation of ANKRD37 in colon cancer. *Exp. Cell Res.* 395, 112175 (2020).

28. Tan, W. *et al.* ANKRD37 inhibits trophoblast migration and invasion by regulating the NF‐κB pathway in preeclampsia. *J. Gene Med.* 24, e3416 (2022).

29. DiStefano, M. T. *et al.* The Lipid Droplet Protein Hypoxia-inducible Gene 2 Promotes Hepatic Triglyceride Deposition by Inhibiting Lipolysis*. *J. Biol. Chem.* 290, 15175–15184 (2015).

30. Das, K. M. P. *et al.* Hypoxia-inducible lipid droplet-associated protein inhibits adipose triglyceride lipase. *J. Lipid Res.* 59, 531–541 (2018).

31. VandeKopple, M. J. *et al.* HILPDA Regulates Lipid Metabolism, Lipid Droplet Abundance, and Response to Microenvironmental Stress in Solid Tumors. *Mol. Cancer Res.* 17, 2089–2101 (2019).

32. Lee, M.-J., Kim, J.-Y., Suk, K. & Park, J.-H. Identification of the Hypoxia-Inducible Factor 1α-Responsive HGTD-P Gene as a Mediator in the Mitochondrial Apoptotic Pathway. *Mol. Cell. Biol.* 24, 3918–3927 (2004).

33. Yoneda, T., Sato, M., Maeda, M. & Takagi, H. Identification of a novel adenylate kinase system in the brain: Cloning of the fourth adenylate kinase. *Mol. Brain Res.* 62, 187–195 (1998).

34. Lanning, N. J. *et al.* A Mitochondrial RNAi Screen Defines Cellular Bioenergetic Determinants and Identifies an Adenylate Kinase as a Key Regulator of ATP Levels. *Cell Rep.* 7, 907–917 (2014).

35. Kong, F., Binas, B., Moon, J. H., Kang, S. S. & Kim, H. J. Differential expression of adenylate kinase 4 in the context of disparate stress response strategies of HEK293 and HepG2 cells. *Arch. Biochem. Biophys.* 533, 11–17 (2013).

36. Liu, R. *et al.* Enzymatically inactive adenylate kinase 4 interacts with mitochondrial ADP/ATP translocase. *Int. J. Biochem. Cell Biol.* 41, 1371–1380 (2009).

37. Fujisawa, K. *et al.* Modulation of anti-cancer drug sensitivity through the regulation of mitochondrial activity by adenylate kinase 4. *J. Exp. Clin. Cancer Res.* 35, 48 (2016).

38. Jan, Y.-H. *et al.* Adenylate kinase 4 modulates oxidative stress and stabilizes HIF-1α to drive lung adenocarcinoma metastasis. *J. Hematol. Oncol.* 12, 12 (2019).

39. Brugarolas, J. *et al.* Regulation of mTOR function in response to hypoxia by REDD1 and the TSC1/TSC2 tumor suppressor complex. *Genes Dev.* 18, 2893–2904 (2004).

40. Corradetti, M. N., Inoki, K. & Guan, K.-L. The Stress-inducted Proteins RTP801 and RTP801L Are Negative Regulators of the Mammalian Target of Rapamycin Pathway*. *J. Biol. Chem.* 280, 9769–9772 (2005).

41. Sofer, A., Lei, K., Johannessen, C. M. & Ellisen, L. W. Regulation of mTOR and Cell Growth in Response to Energy Stress by REDD1. *Mol. Cell. Biol.* 25, 5834–5845 (2005).

42. Vega-Rubin-de-Celis, S. *et al.* Structural Analysis and Functional Implications of the Negative mTORC1 Regulator REDD1 ,. *Biochemistry* 49, 2491–2501 (2010).

43. Horak, P. *et al.* Negative feedback control of HIF-1 through REDD1-regulated ROS suppresses tumorigenesis. *Proc. Natl. Acad. Sci.* 107, 4675–4680 (2010).

44. Tirado-Hurtado, I., Fajardo, W. & Pinto, J. A. DNA Damage Inducible Transcript 4 Gene: The Switch of the Metabolism as Potential Target in Cancer. *Front. Oncol.* 8, 106 (2018).

45. Nakamura, Y. *et al.* BNIP3 and NIX Mediate Mieap-Induced Accumulation of Lysosomal Proteins within Mitochondria. *PLoS ONE* 7, e30767 (2012).

46. Chen, G. *et al.* The E1B 19K/Bcl-2–binding Protein Nip3 is a Dimeric Mitochondrial Protein that Activates Apoptosis. *J. Exp. Med.* 186, 1975–1983 (1997).

47. Bruick, R. K. Expression of the gene encoding the proapoptotic Nip3 protein is induced by hypoxia. *Proc. Natl. Acad. Sci.* 97, 9082–9087 (2000).

48. Bhattacharya, S. *et al.* Functional role of p35srj, a novel p300/CBP binding protein, during transactivation by HIF-1. *Genes Dev.* 13, 64–75 (1999).

49. Berlow, R. B., Dyson, H. J. & Wright, P. E. Hypersensitive termination of the hypoxic response by a disordered protein switch. *Nature* 543, 447–451 (2017).

50. Aprelikova, O., Wood, M., Tackett, S., Chandramouli, G. V. R. & Barrett, J. C. Role of ETS Transcription Factors in the Hypoxia-Inducible Factor-2 Target Gene Selection. *Cancer Res.* 66, 5641–5647 (2006).

51. Liu, T. *et al.* Hypoxia-induced PLOD2 promotes clear cell renal cell carcinoma progression via modulating EGFR-dependent AKT pathway activation. *Cell Death Dis.* 14, 774 (2023).

52. Slot, A. J. van der *et al.* Identification of PLOD2 as Telopeptide Lysyl Hydroxylase, an Important Enzyme in Fibrosis*. *J. Biol. Chem.* 278, 40967–40972 (2003).

53. Kang, H. *et al.* The HIF-1α/PLOD2 axis integrates extracellular matrix organization and cell metabolism leading to aberrant musculoskeletal repair. *Bone Res.* 12, 17 (2024).

54. Thorens, B. & Mueckler, M. Glucose transporters in the 21st Century. *Am. J. Physiol.-Endocrinol. Metab.* 298, E141–E145 (2010).

55. Hayashi, M. *et al.* Induction of glucose transporter 1 expression through hypoxia-inducible factor 1α under hypoxic conditions in trophoblast-derived cells. *J. Endocrinol.* 183, 145–154 (2004).

56. Yasumoto, K., Kowata, Y., Yoshida, A., Torii, S. & Sogawa, K. Role of the intracellular localization of HIF-prolyl hydroxylases. *Biochim. Biophys. Acta (BBA) - Mol. Cell Res.* 1793, 792–797 (2009).

57. Epstein, A. C. R. *et al.* C. elegans EGL-9 and Mammalian Homologs Define a Family of Dioxygenases that Regulate HIF by Prolyl Hydroxylation. *Cell* 107, 43–54 (2001).

58. Ramakrishnan, S., Anand, V. & Roy, S. Vascular Endothelial Growth Factor Signaling in Hypoxia and Inflammation. *J. Neuroimmune Pharmacol.* 9, 142–160 (2014).

59. Arcondéguy, T., Lacazette, E., Millevoi, S., Prats, H. & Touriol, C. VEGF-A mRNA processing, stability and translation: a paradigm for intricate regulation of gene expression at the post-transcriptional level. *Nucleic Acids Res.* 41, 7997–8010 (2013).

60. Rehn, M. *et al.* Hypoxic induction of vascular endothelial growth factor regulates murine hematopoietic stem cell function in the low-oxygenic niche. *Blood* 118, 1534–1543 (2011).

61. Bernstein, B. E. & Hol, W. G. J. Crystal Structures of Substrates and Products Bound to the Phosphoglycerate Kinase Active Site Reveal the Catalytic Mechanism †. *Biochemistry* 37, 4429–4436 (1998).

62. Li, X. *et al.* Mitochondria-Translocated PGK1 Functions as a Protein Kinase to Coordinate Glycolysis and the TCA Cycle in Tumorigenesis. *Mol. Cell* 61, 705–719 (2016).

63. Grandjean, G. *et al.* Definition of a Novel Feed-Forward Mechanism for Glycolysis-HIF1α Signaling in Hypoxic Tumors Highlights Aldolase A as a Therapeutic Target. *Cancer Res.* 76, 4259–4269 (2016).

64. Holmquist-Mengelbier, L. *et al.* Recruitment of HIF-1α and HIF-2α to common target genes is differentially regulated in neuroblastoma: HIF-2α promotes an aggressive phenotype. *Cancer Cell* 10, 413–423 (2006).

65. Cabibbo, A. *et al.* ERO1-L, a Human Protein That Favors Disulfide Bond Formation in the Endoplasmic Reticulum*. *J. Biol. Chem.* 275, 4827–4833 (2000).

66. Takei, N. *et al.* Hypoxia-inducible ERO1α promotes cancer progression through modulation of integrin-β1 modification and signalling in HCT116 colorectal cancer cells. *Sci. Rep.* 7, 9389 (2017).

67. Gupta, N. *et al.* ERO1α promotes hypoxic tumor progression and is associated with poor prognosis in pancreatic cancer. *Oncotarget* 10, 5970–5982 (2019).

68. May, D. *et al.* Ero1-Lα plays a key role in a HIF-1-mediated pathway to improve disulfide bond formation and VEGF secretion under hypoxia: implication for cancer. *Oncogene* 24, 1011–1020 (2005).

69. Koseki, T., Inohara, N., Chen, S. & Núñez, G. ARC, an inhibitor of apoptosis expressed in skeletal muscle and heart that interacts selectively with caspases. *Proc. Natl. Acad. Sci.* 95, 5156–5160 (1998).

70. Ekhterae, D. *et al.* ARC Inhibits Cytochrome c Release From Mitochondria and Protects Against Hypoxia-Induced Apoptosis in Heart-Derived H9c2 Cells. *Circ. Res.* 85, e70-7 (1999).

71. Ao, J., Kuang, L., Zhou, Y., Zhao, R. & Yang, C. Hypoxia-inducible Factor 1 regulated ARC expression mediated hypoxia induced inactivation of the intrinsic death pathway in p53 deficient human colon cancer cells. *Biochem. Biophys. Res. Commun.* 420, 913–917 (2012).
