# Supplementary material for: Differential roles of the type I and II secretion systems for the intracellular ABC141 Acinetobacter baumannii infection, which elicits an atypical hypoxia response in endothelial cells
Source: PLoS Pathog. 2026 Feb 9;22(2):e1013265. doi: 10.1371/journal.ppat.1013265 (PMC12912691; doi:10.1371/journal.ppat.1013265)
Supplement: Attachment — Submitted filename: Response to the reviewers.pdf [file ppat.1013265.s016.pdf]

Dear Editors and Reviewers,

Please find below our point-by-point answer to all the comments.
We thank you all for the constructive feedback and excellent suggestions for additional experiments.

Kind regards
Suzana Salcedo

Editor Comments:

The conclusions of your findings of your study would be further strengthened by addressing two key points: 1) Is the growth of cell-invasive and non cell-invasive Acinetobacter species equally affected by hypoxia?
To address this point, we have tested all the strains in our possession capable of intracellular multiplication in non-phagocytic cells and a few additional strains that cannot invade and/or multiply inside cells. We have found no correlation between these phenotypes and ability to grow in hypoxia conditions. For example, for invasive and "intracellular" strains we observed strain C4 and ABC020 do not efficiently grow in hypoxia, whereby strain R10 does. In contrast, strains previously reported as not able to survive within host cells R141 and AB5075 cannot grow efficiently in hypoxia whereby ABC056 and AIDS020 can. The implications of these data and the limitations of this analysis are now discussed in the manuscript (lines 281-289; 408-411) and the data included as Supplementary Figure 5.

2) whilst the scarcity of effective probes detecting ammonia is hurdle that limits the experimental validation of the data, other evidence (including those currently not included in the present manuscript) would further support the conclusions of the manuscript, e.g. does strain 398 replicate in endothelial cells?
The new data are presented in Figures 2 and Supplementary Figure 1. Ab398 did not efficiently multiply within endothelial cells with only very few cells showing higher numbers of intracellular bacteria (Figure 2D). Conversely, ABC141 did not efficiently multiply in RAW macrophage-like cells, unlike Ab398 (Figure 2D). However, it is essential to note that the strain Ab398 is very poorly invasive in endothelial cells (less than 1% of cells show intracellular bacteria at 2h post-infection – Supplementary Figure 1B). We have discussed these results and their interpretation in the revised manuscript (lines 154-158; 375-376).

Reviewers' Comments:

Reviewer #1: 1. The authors mention preliminary data not included in the manuscript that ABC141 cannot multiply within macrophages and that Ab398 does not secrete ammonia in endothelial cells (lines 354-359). Preferably, these data should be included, as they

represent cross-experimental controls for interpretation of the NH3 data shown for ABC141 in endothelial cells in Figure 2C and for AB398 and 19606 in RAW cells in Figure S1. Related, it may be helpful to clarify the phenotypes for A. baumannii C4 previously reported by the authors, e.g. ACV acidification and ammonia production, etc.

We have now included the multiplication data for ABC141 and Ab398 in both endothelial and RAW macrophage-like cells in Figure 2D and Supplementary Figure 1B. Ab398 did not efficiently multiply within endothelial cells with only very few cells showing higher numbers of intracellular bacteria (Figure 2D). However, Ab398 is very poorly invasive in endothelial cells with less than 1% of cells show intracellular bacteria at 2h post-infection (Supplementary Figure 1B). Conversely, ABC141 did not efficiently multiply in RAW macrophage-like cells, unlike Ab398 (Figure 2D). Therefore, there is no correlation between ability to produce ammonia and intracellular growth (lines 154-158; 375-376).

We have not extended this study for C4 as we do not have RNASeq data for this strain. We are currently applying for funding to expand to other strains and setting up a high-content screen for a broader collection. We therefore decided to keep this manuscript focused on ABC141.

Reviewer #2: The authors note in Supp. Fig 2A and line 396 of the discussion that bacterial expression changes in the dual-RNA-seq were being compared to that of inocula grown in rich media (LB). Is it possible that iron and zinc responsive transcriptional changes were in fact due to down-shift in the nutrient availability of the media used to propagate the endothelial cell line? As a control for this possibility, the authors could perform qRT-PCR to quantify bacterial transcription of zinc and iron uptake systems within the host cell relative to a tissue culture media only control.

We agree that this is a possibility and indeed mentioned this limitation in the discussion. In our experimental set up we are comparing inoculum (grown in LB) with bacteria at 24h inside the cell. RNA turnover is quite fast, and the vacuole went through many modifications during the 24h period. Therefore, the ideal experiment is to compare the RNAseq of the early vacuole in the first minutes of infection, an intermediate vacuole and the replicative vacuole. Unfortunately, we did not obtain enough RNA from bacteria at these early timepoints for the Dual-RNASeq. In retrospect, a single cell RNA approach would have been better. However, it is our opinion that the DMEM with serum (media used for the inocula) does not provide a good control either as it is of a very different composition to the vacuole formed upon invasion.

In addition, all the genes upregulated by qPCR should be tested by qPCR as other responses could be due to the media change. In the case of zinc for example, TPM values for znuD (>100) and D2 (>500) are well above background consistent with an upregulation. Furthermore, a similar study in macrophages using DMEM as control for the DualProteomic reported similar upregulation for zinc related genes and other metals (Janet-Maitre *et al.* BioRXiv 2025). The response to low nutrients and metals in the phagosome is neither new nor surprising.

Reviewer #3: 1. Fig 1 data: it is appropriate to run a time course analysis of the markers shown in panel A (as done in panel for the v-ATPase). It will be appropriate to add an additional graph in which colleagues do show the number of infected cells over time, and the number of bacteria per infected cell over time.

The number of infected cells over time and number of bacteria over time was previously published in the original study describing the ABC141 strain as well as the kinetics for the main marker LAMP1 (Rubio *et al.* 2025). The aim of our analysis was to confirm that the typical markers of early and late endosomes are present at the different stages of the vacuole biogenesis, as expected and described for most pathogens interacting with the endocytic pathway. We chose to only quantify V-ATPase, Lysotracker and Lysoview as their kinetics are essential for concluding on the acidification of this compartment.

2. It will be appropriate to test the fate of UV-killed bacteria; it is anticipated that they will reside in a classical phagolysosome hence providing a positive control for same the analysis. If still this is not the case, then this is a remarkable finding demonstrating that a surface component is implicated in the maturation of the ACV. Follow up simple experiments testing a capsule, and LPS O-polysaccharide mutant may establish the relative contribution of any of these polysaccharides. If none of them are implicated, then these observations will open the avenue for future studies to run a comprehensive screen (this screen is beyond the scope of this study).

Unfortunately, heat-killed bacteria are not taken up by endothelial cells, which are poorly phagocytic. Defining the mechanisms implicated in entry and the contribution of surface components is beyond the scope of this manuscript and is one of the main research topics currently being investigated in the lab.

3. The egress of the ACV is one of the most novel observations of this study. Colleagues should clarify whether egressed bacteria are still protected within the ACV, and if this the case whether cells uptake ACVs.

This is an important question that is difficult to address. We carried out a simple experiment by plating the supernatant before and after treatment with colistin. Data are shown in Figure A of this letter. No bacteria were detected after antibiotic treatment suggesting they may not be enclosed within a vacuole.

However, this experiment is difficult to interpret as the vacuolar membrane may be fragilized during egress and permeable to the antibiotic. We have not detected any egressed intact vacuoles containing bacteria but are uncomfortable reporting this as a conclusive result until we can image the egress step with live cell imaging, which we have not yet succeeded in doing.

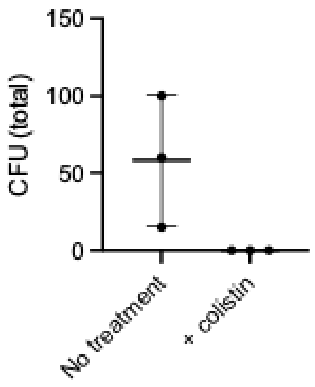

Figure A. Cells were infected for 48h, washed, and supernatants collect at 72h post-infection. Half of the samples were then treated with colistin at 25 µg/ml for 1h and then plated for CFU enumeration.

4. Is the acidification of the ACV essential for intracellular survival?
To address this, we analyzed ABC141 multiplication inside cells in the presence of Bafilomycin (added at 2h post-infection), which inhibits vATPase and prevents lysosomal acidification. ABC141 intracellular multiplication increased in the presence of bafilomycin, suggesting that the acidification is detrimental for *A. baumannii*. The data are now included in Figure 2E and the text modified accordingly (lines 159-162).

5. Colleagues may need to confirm whether all ACVs are egressed or whether there are two types of vacuoles: ones that do remain and it is where Acinetobacter multiplies, and those that are egressed. If there are two types of vacuoles is there any marker suitable to identify them?
This is an excellent point that we do not have the tools to address. We do not know if there are multiple types of vacuoles mediating egress nor have markers to differentiate them. We are confident there is a proportion of vacuoles that do not have multiplying bacteria as the number of bacteria per vacuole at 24h can range from 1 to above 20. We have not characterized the non-replicating vacuoles extensively but at 24h they show all the markers we identified thus far to be present on the replicating vacuoles, so we cannot distinguish them.

6. Colleagues may wish to assess Hif1a activation by immunoblotting. This will add further evidence to the microscopy work.
We have included the western blot (Figure 3E).

7. Fig 4G: do the mitochondria colocalize with ACV or they are just in proximity? without any quantitative measurement it is difficult to sustain the notion of "mitochondria accumulation"
This is a very valid point. The text has been modified for clarity (lines 259 and 401 and 404/405) and refer to proximity.

Reviewer #4: Major points

- The authors report induction of a hypoxia response upon intracellular infection. Although the transcription of genes involved in hypoxia was increased during infection, the authors could not detect hypoxia using available fluorescent probes. The authors show decreased growth of ATCC19606 in hypoxic conditions, however, ABC141, which grows slower according to figure 5A, doesn't seem to be impacted by hypoxia. It is possible that ABC141 has evolved the capacity of switching its metabolism to grow under hypoxia. How

does that translate to other invasive and noninvasive strains? Testing growth in hypoxia of other invasive and non-invasive strains would support the idea that growth in hypoxia is required for intracellular replication.
We have tested an additional 4 strains capable of intracellular growth by measuring the OD and also confirming by CFU counts. The ability to adapt to hypoxia is not correlated to the intracellular multiplication, with C4 and ABC020 not capable of adapting to hypoxia, BMBF_193 growth also significantly impacted and only R10 still showing unaffected growth. Furthermore, several strains that do not multiply inside cells were also able to grow in hypoxic conditions. These data are included as Supplementary Figure 5 and the text modified accordingly (lines 281-289; 408-411).

- ABC141 does not produce ammonia in endothelial cells. Is this specific to this strain or would this happen to any strain in the ACV within endothelial cells? Is ABC141 less capable of producing ammonia than 398? Or in general, Ab strains do not produce ammonia within endothelial cells, possibly due to a metabolic switch towards fatty acid utilization or other carbon sources rather than amino acids. This is alluded in the discussion with some data not included in the manuscript, indicating that ABC141 does not replicate in macrophages. Is 398 able to replicate in endothelial cells? Would produce ammonia then? It would be important to clarify so we can conclude about the role of ammonia in replication within endothelial cells
Unfortunately, we cannot obtain a reliable readout for Ab398 ammonia production in endothelial cells as less than 1% of cells get infected with this strain. Of the few bacteria that are taken up, they do not efficiently multiply. In contrast, ABC141 does not significantly multiply in RAW macrophage-like cells. This prevents us from doing a direct comparison between these strains in infected cells. All data is now included in Figure 2D and Supplementary Figure 1B. The text has been modified accordingly (lines 154-158; 375-376).

- Transcriptomic data was obtained comparing RNA from intracellular bacteria and late stationary phase bacteria grown in LB. This is probably not the best comparison. No more work is needed, but this limitation needs to be added in the discussion.
This limitation was already mentioned in the discussion (lines 420-424).

Part III – Minor Issues: Editorial and Data Presentation Modifications
Reviewer #1: 1. It is not clear the rationale for why the EA.hy929 cell line was used (Line 135). It would strengthen the authors' conclusions to test the findings in other cell lines, especially primary cells, since the EA.hy929 cell line is a rather unique hybrid cell line; however, the conclusions made are well-supported and the limitations are discussed.
We agree that primary endothelial cells models would be important to test and mentioned this in the discussion. Ideally, proper 3D cultures need to start being used for all studies. This is beyond the scope of this manuscript.

2. Lines 233-234: "Detectable"… "was detected" might be typo

Corrected

3. Data is presented in the discussion that "a significant up-regulation of an anti-sense long non-coding RNA (lncRNA) HIF1A-AS was observed (over ten-fold)" (line 371-372). I could not find HIF1A in Table S1 and it would be helpful to state the fold change more precisely as in the rest of the manuscript. HIF1A-AS3 was in Table S1 and appears to be upregulated 40-60-fold, the authors could consider discussing the implications of HIF1A-AS3 upregulation
HIF1A is in Table S1 as ENSG00000100644. The lncRNA are now all grouped under the nomenclature HIF1A-AS3 as a single locus with the most recent annotation that we used. In the discussion we were referring to the increase in HIF1A-AS3. We have corrected the typo and added the precise fold increases (lines 393-399). Apologies for the confusion.

4. Line 399-400: There could also be contributions to OM zinc passage by other transporters or metallophore/TonB-dependent transporter systems, which sometimes have promiscuity for multiple metals. This possibility was not addressed, e.g. by determining whether the ΔznuD1 ΔznuD2 mutants have a growth defect in media without zinc.
This is an interesting point. To test this possibility, we analyzed the growth curve of the mutant in media depleted of zinc using a well-established zinc chelator TPEN as previously done in the context of Znu genes in *A. baumannii* ATCC 17978 (Hesse *et al.* 2019). Both the wild-type and the mutant showed decreased growth in zinc chelating conditions a phenotype that was reversed by adding zinc. Importantly, the mutant lacking *znuD1znuD2* showed lower growth levels in the absence of zinc, confirming its role as a zinc transporter in ABC141(Supplementary Figure 8A and B). However, the *ΔznuD1 ΔznuD2* mutant was still able to grow, consistent with other systems potentially being involved in zinc transport in starved conditions. Even at higher concentration of TPEN the mutant was still capable of exponential growth (Supplementary Figure 8C and D). The text was modified accordingly, including in the discussion (lines 330-333; 428-431).

5. Lines 513-527, 586-598.: When discussing counting bacteria and cells, the method should be clarified. E.g. were the cells counted manually or by a computer software? If counted manually, were they blinded? What intensity of signal was considered "Cell Tracker Green positive?"
These were all clarified in the methods (lines 645; 577).
For this study, all counts for multiplication were done manually. We really need to develop an automated image analysis for the intracellular multiplication!

6. Table 3: The targets and host species of the secondary antibodies should be clarified (i.e. Alexa-Fluor 488 goat anti-rabbit) and the product number should be provided for primary antibody anti-H4A3.
The product name in the DSHB (Developmental Studies Hybridoma Bank) is the name of the clone H4A3, which was added. The species and target host were clarified.

7. Table 3 and line 499: The red-549-mAb with the Hypoxyprobe seems to be a primary antibody because it is not binding another antibody.
Done

8. Figure S4: It would be helpful to reformat as a table with text instead of a screenshot so that readers can easily copy, paste, and search GO terms.
A supplementary new Table S2 was uploaded to facilitate analysis.

9. There are minor formatting issues in the references, such as missing species italicizations.
Thanks.

Reviewer #2: 1. In Figure 7C, were comparisons made with WT at every time point and with both mutant strains? If so please indicate the results of those statistical comparisons on the figure.
It is not clear to us why all comparisons are relevant. We added the main *gspD* ones. All additional comparisons are in Table S5.

2. It is noted in Table 1 that ABC141 is an invasive skin isolate, whereas Ab398 was isolated from urine. If the authors believe that the source of host tissue/infection is potentially relevant to the differences observed in their intracellular phenotypes, this information could be added to the discussion section after line 356 to further bolster the discussion point that A. baumannii isolates may have evolved different strategies for propagating in different host niches.
It would be unwise to speculate based on two strains, but a sentence was added to highlighted this may be an interesting avenue for future work (lines 379-380).

Reviewer #3: 1. Colleagues need to provide a description on the methods section on the criteria followed to determine whether the ACV is positive or not for any of the markers assessed in this work.
Done (lines 638-640).

2. For all the microscopy analysis, colleagues need to indicate exactly how many ACVs (or infected cells when needed) were analysed in each group/time point 50 ACVs per condition seems a rather low number if 3 independent experiments were run.
A minimum of 50 ACVs per condition and per experiment were quantified for the multiplication. We have added all the vacuoles counted as Table S6.

3. Is there any role for the T6SS in intracellular survival?
ABC141 does not encode for T6SS.

4. Colleagues may wish to explain better why they have chosen to further investigate

bacterial systems that they do not seem to be picked up by the dual RAN seq analysis as DGEs.
Because they were shown by others as relevant for *A. baumannii* virulence.

5. There is no good correlation between the ACVs shown in most of the beautiful confocal images (containing more than 6-8 bacteria as average) and the data shown in Fig 6D and Fig 7C in which the average is 2 bacteria per ACV within the first hours. This review feels that there maybe two types of vacuoles (please refer to point 5 part II).
This is the reason we present all data points showing clearly that there are vacuoles with more than 10-20 bacteria and many vacuoles with few. If we are showing images of vacuoles suited for multiplication (the focus of this study), we select those with at least 5 bacteria which corresponds to our definition of a vacuole with replicating bacteria.
It is very likely that not all bacteria are multiplying, and some may simply be persisting. We agree with the reviewer that several types of vacuoles are present. Hopefully, future live cell imaging experiments will help decipher this.

Reviewer #4: Minor points
- Could the authors elaborate on what could be the source of hypoxia?
Not really; we are very intrigued by this phenotype. If the reviewer has suggestions, we would be grateful to hear them. It may be a simple stress response induced upon bacterial infection and not necessarily due to lack of oxygen.

- The authors show that vacuoles are acidic but non degradative. Could they speculate on how this could be achieved?
The transition between late endosomes and lysosomes is a continuum. It is possible they are acidic but still more of a late endosome. Until we can measure precise intra-vacuolar pH we cannot address this. Our data support that these vacuoles are not bonafide lysosomes as they lack active degradative enzymes as stated in the manuscript.

- Tables and figures show Log2(FC) whereas the text mentions fold change. Consistency would ease the reading.
In response to a comment by reviewer #1, we included now more fold-changes in the text. Respectfully, we believe it to be more intuitive to understand compared to the log2(FC) values, which are more appropriate for some types of plotting (e.g. the volcano plots) but are otherwise less easy to grasp. This is standard practice for reporting RNA-seq data, and we would prefer to leave it as it is.

- Figure 3A, indicating the legend for the black dots on the volcano plots would we helpful.
The information was added in the legend. Black dots correspond to genes previously identified as linked to hypoxia signaling pathways.

- Normoxia is stated as 21% in the text but 20% O2 in the figures.
This has been corrected to 21%.

- The scale on fig. 5A is unclear, from 0- 5X 107

The scale was for $5x10^7$ CFU/ml per tick increments. We have converted to Log scale to make it easier.

- Fig 7C, the authors could adda dotted line or any other detail on the graph to guide the reader on the decreased number of bacteria per vacuole in a dhlyD mutant

Added

- Line 354 – Alluded in "major points". This preliminary data is important and should be included in the manuscript.

Added.

Figures

- Legend Fig1 - Dapi was used to visualize nuclei and bacteria - '(blue)' should be added

Corrected.

- DAPI should be capitalized

All have been modified

- Fig3B – for infection with ABC141 / 20% - 'O2' is missing

Corrected.

- *Fig3 legend – the time at which B) images were taken should be added as well as the meaning of '***'*

Done.
