# Supplementary material for: Differential roles of the type I and II secretion systems for the intracellular ABC141 Acinetobacter baumannii infection, which elicits an atypical hypoxia response in endothelial cells
Source: PLoS Pathog. 2026 Feb 9;22(2):e1013265. doi: 10.1371/journal.ppat.1013265 (PMC12912691; doi:10.1371/journal.ppat.1013265)
Supplement: Attachment — Submitted filename: Response to the reviewers 2.pdf [file ppat.1013265.s017.pdf]

Dear Editors and Reviewers,

We have carried out all the modifications requested:

1. included a new paragraph in the discussion:

Although we conducted a detailed characterization of the intracellular adaptation of ABC41 in endothelial cells, many questions remain to be addressed. As mentioned above, key aspects of this study should be expanded to other *A. baumannii* strains and other cell types, notably in primary human cells and in 3D cell culture models that better mimic human tissues. The nature of the newly identified vacuole that enables ABC141 egress also remains unclear. Do these vacuoles arise from the replicative compartments, or do they constitute a distinct specialized compartment? The discovery of specific markers for these egress competent vacuoles is needed to answer these questions. The role of the secretion systems in this process is also of interest, but these egress vacuoles may also result from a cell-intrinsic defense mechanism in response to the failure to degrade vacuole contents in the endosomal and autophagic pathways. Their contribution to tissue colonization, bacterial dissemination and host pathology needs to be investigated *in vivo.*

2. Added the curves with Zinc in Supp Fig 8.

We have also noted a mistake in Figure 2D in the kinetics, which was wrongly labeled as 3 and 24h for both cell types and should have been 2 and 24 for endothelial cells and 2 and 6h for RAW macrophages. We have included 2, 6 and 24h data for both cells for clarity.

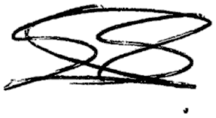

Kind regards
Suzana Salcedo
